# Correction: Aptamer AS1411 interacts with the KRAS promoter/hnRNP A1 complex and shows increased potency against drug-resistant lung cancer

**DOI:** 10.1039/d4md90013h

**Published:** 2024-04-15

**Authors:** Yuejie Zhu, Xiang Li, Qi Zhang, Xiantao Yang, Xudong Sun, Yi Pan, Xia Yuan, Yuan Ma, Bo Xu, Zhenjun Yang

**Affiliations:** a State Key Laboratory of Natural and Biomimetic Drugs, School of Pharmaceutical Sciences, Peking University Beijing 100191 China yangzj@bjmu.edu.cn +86 10 82802503 +86 10 82802503; b School of Pharmacy, Chengdu Medical College 783 Xindu Avenue, Xindu District Chengdu 610500 China

## Abstract

Correction for ‘Aptamer AS1411 interacts with the KRAS promoter/hnRNP A1 complex and shows increased potency against drug-resistant lung cancer’ by Yuejie Zhu *et al.*, *RSC Med. Chem.*, 2024, https://doi.org/10.1039/d3md00752a.

The authors regret that the title of their manuscript showed an error. “AS411” should be “AS1411”. The correct title is shown above.

The Royal Society of Chemistry apologises for these errors and any consequent inconvenience to authors and readers.

## Supplementary Material

